# Anesthetic management for a patient with myotonic dystrophy with remimazolam

**DOI:** 10.1186/s40981-021-00413-3

**Published:** 2021-01-12

**Authors:** Yasuhiro Morimoto, Aya Yoshimatsu, Manabu Yoshimura

**Affiliations:** Department of Anesthesia, Ube Industries Central Hospital, 750 Nishikiwa Ube, Yamaguchi, 755-0151 Japan

**Keywords:** Flumazenil, Myotonic dystrophy, Remimazolam

## Abstract

**Background:**

Patients with myotonic dystrophy may have increased sensitivity to drugs used for anesthesia. We successfully managed general anesthesia in a patient with myotonic dystrophy using a novel intravenous anesthetic, remimazolam.

**Case presentation:**

The patient was a 46-year-old man, 169 cm in height, and weighing 60 kg. He was diagnosed with myotonic dystrophy 5 years previously. Phacoemulsification for both eyes was scheduled under general anesthesia. Anesthesia was induced with remimazolam 6 mg/kg/h for 1 min and maintained by continuous infusion at 0.25 mg/kg/h during surgery, a 1/4 dose of the standard infusion rate, as indexed by a bispectral index (BIS). Six minutes after remimazolam discontinuation, the patient opened his eyes on verbal command with sufficient spontaneous respiration. Flumazenil (0.2 mg) was administered to boost the patient’s recovery.

**Conclusion:**

In addition to the short-acting anesthetic remimazolam, the presence of the antagonist flumazenil enabled complete recovery from anesthesia, without postoperative complications.

## Background

Myotonic dystrophy which is characterized by myotonia and extra muscular features, including cataracts, cardiac conduction abnormalities, and dysphagia is a rare but serious inherited disorder that may pose substantial problems for anesthetic management [1–3]. Patients with myotonic dystrophy have increased sensitivity to drugs used in anesthesia, such as hypnotics, neuromuscular blocking agents, and opioids. The use of inhalational anesthetics might produce shivering that can precipitate myotonia. Therefore, total intravenous anesthesia (TIVA) is commonly used in these patients. Propofol, commonly used as an anesthetic for TIVA, was suggested for prolonged recovery after anesthesia [2] and respiratory failure [3].

Remimazolam is a novel benzodiazepine that was approved as a general anesthetic in Japan [4]. Remimazolam is rapidly hydrolyzed and metabolized primarily by carboxylesterase in the liver. As the context-sensitive half-time (CSHT) of remimazolam is shorter than midazolam, it has the potential to be a primary intravenous anesthetic for general anesthesia. We successfully managed general anesthesia in a patient with myotonic dystrophy using a novel intravenous anesthetic, remimazolam.

## Case presentation

The patient was a 46-year-old man, 169 cm in height, and weighing 60 kg. He was diagnosed with myotonic dystrophy type 1 5 years previously, with weakness in the distal muscles and electrophysiological tests. The patient complained of vision disability due to cataracts, and phacoemulsification and intraocular lens implantation for both eyes were scheduled under general anesthesia.

Serum creatine phosphokinase level was 2921 U/L (normal range, 43–272 U/L). There were no further abnormal findings on preoperative tests, including an electrocardiogram and transthoracic echocardiogram. Although the relationship between myotonic dystrophy and malignant hyperthermia is controversial, we planned to use total intravenous anesthesia in this case.

The use of propofol, which is a commonly used intravenous anesthetic, might cause prolonged recovery after anesthesia in patients with myotonic dystrophy [2]. Therefore, we planned to use remimazolam to achieve fast recovery in this case.

**Fig. 1:**
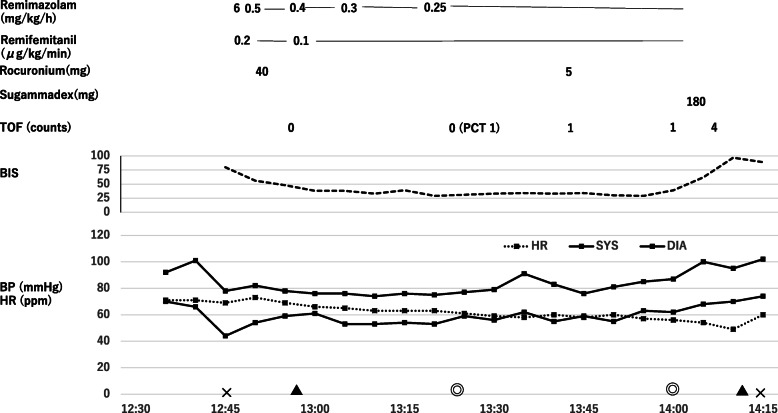
Anesthesia record. TOF train of four, PTC post tetanic count, BIS bispecral index,  BP blood pressure, HR heart rate, SYS systolic blood pressure, DIA diastolic blood pressure, × start of anesthesia or end of anesthesia, ▲ tracheal intubation or extubation, and ◎ start of surgery or end of surgery

**Fig. 2:**
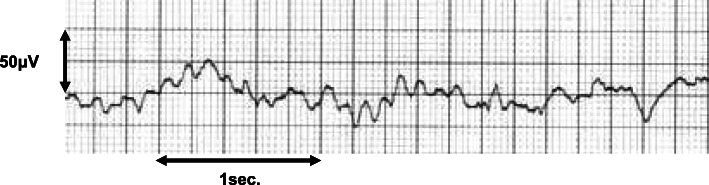
Electro-encephalogram recorded by bispectral index during the surgery. The infusion rate of remimazolam was 0.25 mg/kg/h, and remifentanil was 0.1 μg/kg/min at the BIS value of 34

No premedication was administered. We administered 6 mg/kg/h of remimazolam intravenously for 1 min. The patient lost the response to the verbal command after the infusion of remimazolam (Fig. 1). Continuous infusion of remifentanil (0.2 μg/kg/min) and remimazolam (0.5 mg/kg/h) was started. Neuromuscular monitoring of the left ulnar nerve was commenced using a train-of-four (TOF) stimulus (TOF watch, MSD). Three minutes after administration of 40 mg rocuronium, all four twitch responses disappeared, and tracheal intubation was performed. During surgery, the remimazolam dose was planned to maintain a bispectral index (BIS) value between 40 and 50 with continuous remifentanil infusion (0.1 μg/kg/min). However, the BIS value remained below 40 at a continuous infusion rate of 0.25 mg/kg/h, a 1/4 dose of the standard infusion rate. At this dose, the electroencephalogram showed continuous alpha wave spindles and delta waves (Fig. 2). Therefore, we judged that an appropriate anesthesia level was achieved. An additional 5 mg rocuronium was administered 60 min after the first dose because the first twitch response was observed. The surgery time was 46 min, and the infusion of all anesthetics was stopped. At the end of the procedure, TOF showed the first twitch response, and then 180 mg(3 mg/kg) of sugammadex was administered. Three minutes after administering sugammadex, the TOF recovered to 100%. Six minutes after remimazolam discontinuation, the patient opened his eyes on verbal command with sufficient spontaneous respiration. Next, the tracheal tube was removed. Finally, flumazenil (0.2 mg) was administered to boost the recovery, and the postanesthetic course was uneventful. A post-operative interview with the patient was performed on the day and the next day. He had no memory during the surgery.

## Discussion

Myotonic dystrophy is a rare but serious inherited disorder that may pose substantial problems for anesthetic management including the prolonged recovery after anesthesia and post-operative respiratory failure [1]. Therefore, we planned the anesthesia for this patient to avoid the delayed recovery from anesthesia safely.

The CSHT of remimazolam is estimated at 7.5 min, which is equivalent to propofol [5]. Moreover, the anesthetic effect of remimazolam is antagonized by the benzodiazepine antagonist flumazenil. The availability of an antagonist is one of the advantages of remimazolam over propofol.

Based on these factors, we chose remimazolam as the anesthetic in this case. The patient lost consciousness after a 1-min infusion of 6 mg/kg/h of remimazolam. In a previous phase II trial [6], the mean time of the loss in consciousness with 6 mg/kg/h remimazolam was 102 s. In this case, the dose of remimazolam for induction was smaller than the dose in the phase II trial. During the procedure, the infusion rate was approximately 1/4 of the standard infusion rate, as indexed by BIS. These results suggest that the sensitivity to remimazolam in the patient was high; however, the dose of remimazolam was easily titrated with BIS monitoring. The patient recovered consciousness 6 min after discontinuation of remifentanil infusion. The emergence time was almost equivalent to that of CSHT. This suggests that the level of anesthesia during surgery was maintained at nearly twice the waking level and appropriate anesthesia depth was maintained throughout the surgery. Finally, we used flumazenil to increase the arousal level to prevent postoperative complications. There have been no reports of benzodiazepines including remimazolam as the main general anesthetic for patients with myotonic dystrophy. Further experience should be needed to evaluate the usefulness of remimazolam.

For the neuromuscular blocking agents, we used rocuronium and sugammadex in this case. It is recommended to avoid muscle relaxants if possible in patients with myotonic dystrophy because it might produce prolonged muscle weakness. We used rocuronium under neuromuscular monitoring and reversed the effect with sugammadex. Recently, an uneventful recovery from general anesthesia using sugammadex in a patient with myotonic dystrophy was reported [7, 8]. Opioids may induce muscle rigidity; therefore, omitting opioids is recommended. We used a small dose of short-acting opioid remifentanil in this case because case reports suggest that remifentanil can be used safely in patients with myotonic dystrophy [9].

## Conclusions

We believe that in addition to the short-acting anesthetics remimazolam and remifentanil, the presence of the antagonist flumazenil and sugammadex enables complete recovery from anesthesia, avoiding postoperative complications in patients with myotonic dystrophy. The short-acting benzodiazepine remimazolam and the use of an antagonist is a good choice for anesthetic management in high-risk patients, including those with myotonic dystrophy.

## Data Availability

Not applicable.

## Abbreviations

CSHT: Context-sensitive half-time
TOF: Train-of-four
BIS: Bispectral index
TIVA: Total intravenous anesthesia
